# Factors Associated with COVID-19 Vaccine Acceptance in Morocco: Applying the Health Belief Model

**DOI:** 10.3390/vaccines10050784

**Published:** 2022-05-16

**Authors:** Imane Berni, Aziza Menouni, Younes Filali Zegzouti, Marie-Paule Kestemont, Lode Godderis, Samir El Jaafari

**Affiliations:** 1 Cluster of Competency “Health and Environment”, Moulay Ismail University, Meknes 50000, Morocco or aziza.menouni@kuleuven.be (A.M.); y.filalizegzouti@fste.umi.ac.ma (Y.F.Z.); s.eljaafari@umi.ac.ma (S.E.J.); 2 Environment and Health Unit, Department of Public Health and Primary Care, Katholic Universiteit of Leuven, 3000 Leuven, Belgium; lode.godderis@kuleuven.be; 3 Institute for the Analysis of Change in Contemporary and Historical Societies, Université Catholique de Louvain, 1348 Louvain-la-Neuve, Belgium; marie-paule.kestemont@uclouvain.be; 4 IDEWE, External Service for Prevention and Protection at Work, 3001 Heverlee, Belgium

**Keywords:** COVID-19, Health Belief Model, vaccine acceptance, Morocco

## Abstract

To identify factors that influenced Moroccans’ intention to get a COVID-19 vaccine, a cross-sectional survey among a Moroccan sample was conducted based on Health Belief Model constructs. Participants’ sociodemographic characteristics, perceived susceptibility, perceived severity, perceived barriers, perceived benefits, self-efficacy, cues to action, and intention to receive vaccine data were collected and analyzed using a structural equation model (SEM). The survey was completed by 3800 individuals; 57.2% were men, 44.5% were aged 30 to 44, and 44.6% were married. After controlling for confounders, being a woman and having a chronic disease were associated with higher acceptance of the COVID-19 vaccine. The strongest predictor for the intention of receiving a vaccine was participants who were married. Most of the HBM constructs were shown to be significantly associated with vaccine acceptance. Susceptibility and Benefits were the strongest predictors of acceptance of the COVID-19 vaccine (standardized path coefficient, SPC = 0.23), followed by Severity (SPC = 0.22). Conversely, given the negative correlation between barriers and intention (SPC = −0.08), it is necessary to maintain a high level of transparency regarding the vaccines’ safety. Our study provides guidance for an implementation of vaccination strategies, intending to bolster the overall COVID-19 immunization program.

## 1. Introduction

Since its emergence in December 2019, the coronavirus disease 2019 (COVID-19) pandemic has caused catastrophic damage worldwide. As the health issue continues to linger and a sense of pandemic fatigue starts to take hold [1], it appears that the entire world is looking forward to the introduction of a safe and efficient vaccine [2]. In response, more than 50 vaccines for COVID-19 are either in clinical testing or have already been approved for limited use in some countries [3]. As of February 2021, two vaccines have been approved by the Health Ministry of Morocco for the novel Coronavirus [4]. While it seems that the entire globe is anticipating the arrival of safe and efficacious vaccines, the acceptance of the COVID-19 vaccine by people remains unknown.

In this context, a basic understanding of different factors influencing people’s intention to get vaccinated against COVID-19 is crucial to achieving herd immunity and developing effective interventions that promote vaccine acceptance. One such framework is the Health Belief Model (HBM). According to this model, to adopt healthy behavior, individuals need to consider themselves susceptible to the potential disease and perceive it has serious consequences (i.e., perceived threat), believe that certain health behaviors can improve and promote their wellbeing (i.e., perceived benefit), and that the benefits of healthy behaviors outweighed the costs (i.e., perceived barriers), and that taking action would have a benefit (cues to action) [5,6]. Additionally, as COVID-19 vaccines were not generally accessible in Morocco at the time of this study, we focused on the intention to receive COVID-19 vaccines due to their high correlation with the actual uptake behavior [7].

Previous literature has focused on people’s opinions regarding vaccination programs among the general population before introducing the vaccine. However, applying a behavioral model or theories such as the Health Belief Model (HBM) that could predict the acceptance of the COVID-19 vaccine has received less attention. Two studies were found that applied HBM in the general public context [8,9]. Furthermore, there has been no research conducted in North Africa to assess people’s willingness to get vaccinated against COVID-19. In response, the present study provides valuable insights into how the Health Belief Model impacts the Moroccan general public’s intention toward COVID-19 vaccine acceptance.

Overall, this study contributes to improving awareness in two ways. First, it sheds new light on factors that predict people’s intention to receive the COVID-19 vaccine. Second, it provides more empirical data on who is more likely to get a vaccine to help authorities plan the distribution of COVID-19 vaccines.

### Theoretical Framework and Hypotheses Development

Developed by Champion and Skinner [10], HBM is now used as the most popular social–psychological theory for understanding people’s behaviors in various fields. This study proposed a hypothesis and analyzed six main factors that affect the public towards COVID-19 vaccine acceptance. 

Vaccination, like several others health behaviors, is used to avoid adverse health outcomes [11]. Therefore, it is reasonable that people’s perceptions of how severe and likely a disease is to affect them should influence their willingness to receive a vaccine [12]. In the context of COVID-19 vaccination, if individuals believe themselves to be vulnerable to a COVID-19 disease, think that the disease would have significant consequences, and recognize that vaccination will help to minimize susceptibility and severity, they are likely to seriously undertake healthy behaviors that they think will reduce the risk [13]. Nevertheless, lower mortality rates among younger populations may lead to lower perceived risk and hesitation to get a COVID-19 vaccine [14]. Considering this, our first hypothesis was that perceived susceptibility and severity of COVID-19 will be positively related to vaccination intention. 

Perceived benefits and barriers can affect the intention of the general public to get the COVID-19 vaccination; perceived benefits of getting vaccinated were strong predictors with uptake intent, while perceived barriers were negatively correlated with vaccination intention (e.g., seasonal influenza vaccine) [9]. People who consider the vaccines to benefit themselves and their communities held a generally positive view about COVID-19 vaccines [15]. Likewise, the accelerated development of COVID-19 vaccines and cost concerns have been reported as barriers [15]. Our second hypothesis was that perceived benefits and barriers of COVID-19 vaccination are positively associated with a higher COVID-19 vaccine uptake intent.

**Figure 1:**
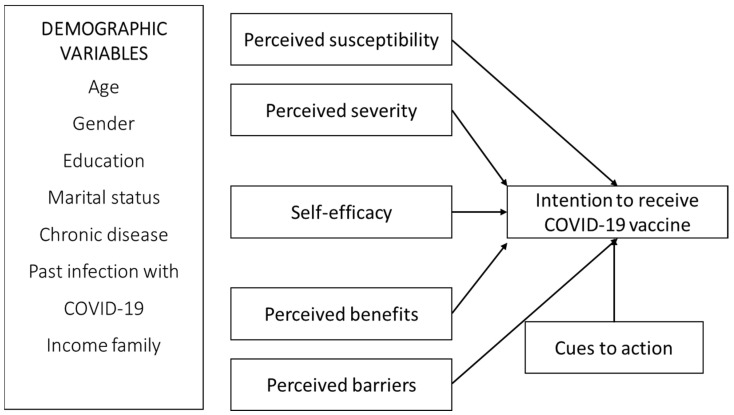
Theoretical framework.

The Health Belief Model (HBM) contains two additional elements: cues to action and perceived self-efficacy [16], as showed on Figure 1. Cues to action work as “triggers” and thus motivate individuals to change behaviors, while self-efficacy is the belief that one can carry out behavior [17]. In the context of the COVID-19 vaccine, providing correct information via the government and experts acts as triggers for promoting engagement in vaccination campaigns [18]. Correspondingly, our final hypothesis was that increased cues to action and self-efficacy would positively affect vaccination intention.

## 2. Methods

### 2.1. Study Design

This cross-sectional questionnaire was conducted from 5 to 20 January 2021, 10 days immediately before the start of the vaccination campaign in Morocco. We decided to collect data online because it was not feasible to perform a community-based national sampling survey during this particular period. Relying on the social network, an online questionnaire was spread via Facebook, WhatsApp, Instagram, Twitter, and LinkedIn using sponsored social network advertisements. We used a convenience sampling method. The eligibility criteria were as follows: (1) subjects aged 18 or above; (2) lived in Morocco. A de-duplication protocol was applied to identify multiple submissions to preserve data integrity, including cross-validation of the eligibility criteria and discrepancies in key data, as well as checking for unusually fast completion time (<15 min). The registered members clicked the link on the platform and responded to the survey voluntarily until the convenience sample covered all 12 regions in Morocco.

Research was conducted with generally accepted ethical principles and approved by the Ethics Committee of Moulay Ismail University (N: CERB-UMI 03/2020). Online consent was obtained from the participants before completing the questionnaire. No compensation was provided.

### 2.2. Participants

A total of 4015 participants clicked on the survey link, and 3970 individuals started the survey, among whom 30 individuals refused to provide informed consent, and 3940 participants provided informed consent and submitted the questionnaire. After excluding 120 participants who did not complete all the questions and 20 respondents younger than 18 years old, the final sample consisted of 3800 participants.

### 2.3. Survey Content

We designed the study instrument after reviewing related questionnaires in published literature [19,20] to incorporate most of the constructs of the Health Belief Model. The first questionnaire section pertained to general information about the participant (age, education, gender, marital status, presence of chronic disease, flu vaccine uptake history, past infection with COVID-19, and monthly household income). The second questionnaire section was designed to measure intention to receive the COVID-19 vaccine on a scale based on the Health Belief Model. Twenty-four items in seven categories were considered. The participants were asked on a Likert scale how strongly they agreed or disagreed with each statement (1 as “strongly disagree” to 5 as “strongly agree”), except for the construct of intention to receive a COVID-19 vaccine, participants indicated for each statement the likelihood that they would consider getting COVID-19 vaccines on a scale of 1 (very unlikely) to 5 (very likely).

The face and content validity of the questionnaire were examined to assure that the items could subjectively/theoretically cover the constructs [21]. For this purpose, a review panel consisting of two specialists at the Faculty of Sciences in Meknes (a professor of biostatistics and epidemiology and a professor of biological sciences) was formed to assess the research instrument. Their feedback was incorporated to enhance the questions’ readability, completeness, and clarity. The questionnaire was piloted on a small sample of participants from the general population prior to the study (*n* = 50). Data from this pilot sample were not used in the subsequent analysis. As a result, the clarity and appropriateness of the questions were evaluated, and the questionnaire was edited accordingly. Additionally, Cronbach’s alpha was employed to examine internal consistency reliabilities of the questions. 

### 2.4. Statistical Analysis

Data analysis was performed using the Statistical Package for Social Sciences (SPSS) version 20 (IBM). Frequencies and percentages were used for the description of the sociodemographic data. The primary outcome variable was a dichotomous variable on acceptance of COVID-19 vaccination. Vaccine acceptance was calculated based on the self-administrated questionnaire. The dependent variable “I intend to get vaccinated” had two possible values: 1 = (“Yes, likely/very likely”); 0 = (“No, very unlikely/unlikely/neutral). Forward univariate analysis based on Pearson’s chi-square tests at a significance level of 5% was used. To determine the association between sociodemographic variables and vaccine acceptance, a multivariable logistic regression analysis was performed. The associations between vaccine acceptance and outcomes are presented as odds ratios (O.R.s) and 95% CIs, after adjustment for confounders that showed the most effect from univariate analysis, including gender, marital status, influenza vaccination status, and infection with COVID-19.

Using the maximum likelihood procedure with AMOS software, Structural Equation Modeling (SEM) was adopted to test the relationship between perceptions and the intention to receive a COVID-19 vaccine. Following Anderson and Gerbing’s [22], a two-stage analytical process was used. First, a measurement model was estimated through Confirmatory Factor Analysis (CFA) by assessing the model fit, convergent validity, discriminant validity, and reliability. Then, a structural model was used to check the best-fitting model.

To assess the fit of the model, various indices were used in the present research, including relative Chi-square (<0.5), GFI (goodness of fit index) (GFI > 0.90), AGFI (adjusted GFI) (AGFI > 0.90), CFI (confirmatory fit index) (CFI > 0.90), IFI (incremental fit index) (IFI > 0.90), RMR (root mean square residual) (RMR > 0.08), and RMSEA (root mean square error of approximation) (RMSEA > 0.08) [23,24]. Regarding the convergent validity, three indicators are evaluated: item loading, average variance extracted (AVE) and construct reliability (CR) [25]. For acceptable convergent validity, item loading and AVE estimates must have a value of 0.5 or greater, while CR estimates must have a value of 0.7 or greater [22,26]. Similarly, discriminant validity was evaluated by comparing the AVE of each construct with the average shared squared variance (ASV) and the maximum shared squared variance (MSV). In this context, AVE should be greater than ASV and MSV [25].

## 3. Results

### 3.1. Descriptive Information of the Sample

Table 1 shows the sociodemographic characteristics of the sample concerning their intention to receive the COVID-19 vaccine. A total of 3800 respondents participated in the survey. Among the survey completers, 42.8% were women, 73.8% were aged 30 to 59, and 57.3% held a Bachelor’s degree or above. Around 55.4% were married, and 42.9% were infected by COVID-19 (Table 1).

About a third of respondents reported intending to vaccinate against COVID-19 (40%). All the sociodemographic variables were significantly associated with the intention to receive the COVID-19 vaccine (Table 1).

### 3.2. Sociodemographic Factors Associated with Acceptance of COVID-19 Vaccine

From the binary logistic regression analysis (Table 2), and after controlling for confounders, being a woman, married, and having a chronic disease was associated with higher acceptance of COVID-19 vaccine (e.g., gender: OR, 1.24; 95% Cl, 1.09–1.42; *p* < 0.001). Compared with uninfected participants, those with confirmed COVID-19 were more likely to get vaccinated (OR, 1.20; 95% Cl, 1.05–1.38; *p* < 0.001). The strongest predictor for intention to receive a COVID-19 vaccine was participants who is married; these participants were significantly more likely than other groups to receive a COVID-19 vaccine (OR, 131; 95% Cl, 1.15–1.50; *p* < 0.05)

### 3.3. Statistics of Key Variables

The descriptive statistics of key variables regarding COVID-19 vaccine acceptance are summarized in Table 3. In the interview results, the construct “perceived barriers” (Bar) showed the highest mean value (mean = 4.53) among all constructs, while the construct “severity of COVID-19” showed the lowest mean value (mean = 3.28) (Table 3). Of the four indicators of the intention to receive the COVID-19 vaccine construct, “I am currently undecided” was the most highly rated.

The construct “self-efficacy” (SE) received a total mean score of 4.5, indicating the Moroccan population’s strong perception of self-efficacy. People seemed to have enough ability to take action for COVID-19 prevention. According to Table 3, the construct “perceived benefits” (Ben) was rated high (mean = 4.5). Most of the respondents had a high level about the indicator “COVID-19 vaccine will be effective in preventing coronavirus.” “Cues to action” also received a high score (mean = 4.31) (Table 3). “Perceived susceptibility” received a moderate score (mean = 3.90). Most respondents felt worried about themselves or their family members contracting the virus.

### 3.4. Predictors of Intention to Vaccinate against COVID-19

#### 3.4.1. Measurement Model

To investigate the adequacy of the model, the full measurement model was estimated by conducting CFA. In this study, the scales used were derived from those used in the relevant literature [24,25]. According to the CFA results (Table 4), all construct standardized factor loadings ranged from 0.419 to 0.901, except for three constructs, and were all significant at *p* < 0.001. Additionally, the AVE and CR values of each construct used in this study were greater than 0.50 and 0.70, respectively, except for AVE of the severity of COVID-19, thereby indicating good convergent validity and reliability. Further, the AVE values were all greater than those of ASV and MSV, supporting discriminant validity. Thus, it can be stated that all model measurements used in this study were valid and reliable.

#### 3.4.2. Structural Model

SEM analysis showed that all fit indices were within acceptable ranges, indicating that the model was well fit to the data (Table 4). The square multiple correlation (R^2^) coefficient of the dependent variable SB was 0.472, which indicated that the investigated constructs explained 47.2% of the variable’s variance in the model.

**Figure 2:**
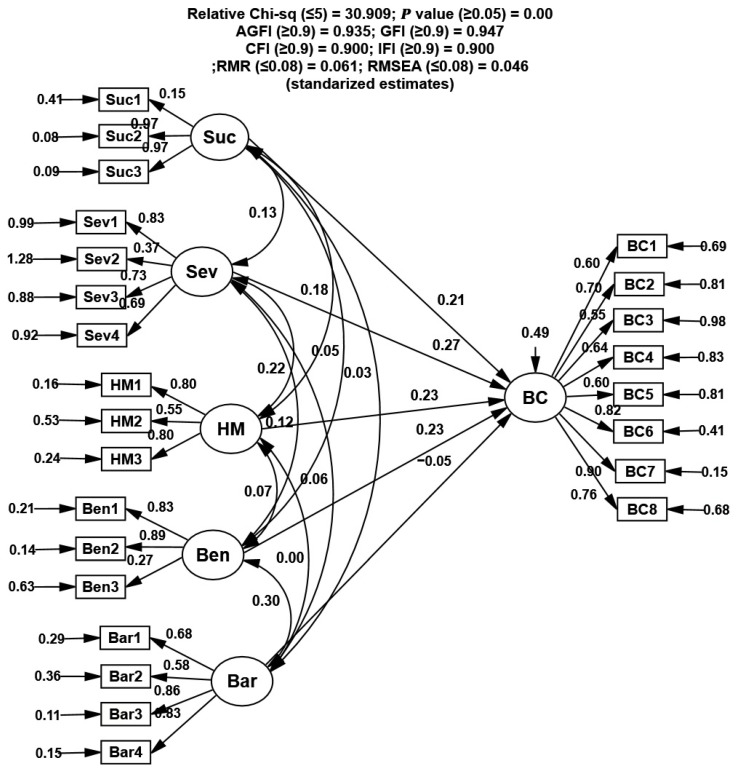
Standardized path coefficients of the factors that directly and indirectly affect intention to receive COVID-19 vaccine in Morocco.

Furthermore, significant associations were found between intention to receive a COVID-19 vaccine and other constructs, namely Sus, Sev, Ben, Bar, SE, and CTA (Figure 2). The perceived susceptibility and perceived benefits were the most important factors influencing intention to get vaccinated in Morocco (SPC = 0.23), followed by perceived severity (SPC = 0.22), cues to action (SPC = 0.17), and self-efficacy (SPC = 0.16). The path analysis of the general public indicated that perceived barriers (SPC = −0.08) had a major negative effect on the intention to receive a COVID-19 vaccine.

Suc—perceived susceptibility; Sev—severity of COVID-19; Bar—perceived barriers; Ben—perceived benefits; SE—self-efficacy; CtA—cues to action; Int—intention to receive COVID-19 vaccine; significant at *p* < 0.001

## 4. Discussion

The current study provides insights into how various psychosocial factors are associated with COVID-19 vaccine uptake among the Moroccan population. The findings indicated that most of the HBM constructs were shown to be significantly associated with vaccine acceptance. In particular, respondents who perceived COVID-19 as serious, believed the vaccine conferred benefits, and received cues to action were significantly more likely to accept the vaccine. On the other hand, the perception of access barriers was negatively associated with their acceptance. Similar to previous research [20,27], we found that the Health Belief Model helps identify major determinants of getting the COVID-19 vaccine. These findings are useful for informing public health strategies to improve willingness and uptake of the COVID-19 vaccine among the general population.

We found a significantly positive correlation between age and intention to receive a vaccine: older participants adopted a higher level in response to COVID-19 vaccine acceptance than younger participants. Malik et al. and Wong et al. [28,29] also reported that vaccine uptake was more common among elderly individuals. Given their greater awareness of the risks associated with coronavirus and their higher risk of contracting the more severe disease and death due to COVID-19 [30], older individuals had higher acceptance of the COVID-19 vaccine. This could also explain, in our findings, why participants with chronic diseases were considerably more likely to express vaccine acceptance. In developed countries, as in developing countries such as Morocco, it is essential to regard these individuals as a top-priority group to receive the COVID-19 vaccine.

Our study corroborates research on other viruses that found that women had higher acceptance of the COVID-19 vaccine [31]. In the USA, it has recently been shown that more women receive COVID-19 vaccines than men, even though more men die of the disease [32]. However, misinformation and access challenges have increased inequities in distribution in most Indian states, and women have received fewer vaccines than men [33]. Vaccine safety has been mentioned as a source of concern [34], and indeed our analyses suggest that women can be more easily convinced than men to get vaccinated.

Past infection with COVID-19 was one of the factors affecting vaccine acceptance. Those with confirmed COVID-19 were more likely to get vaccinated than uninfected people. This is partially consistent with previous research on H1N1 [35]. Many of the respondents of our study experienced COVID-19 disease. Consequently, they thought that the disease could be difficult to control.

Despite the importance of susceptibility and severity of COVID-19 as a significant predictor of vaccine acceptance, the study results revealed that these components had a significant effect on the intention to receive vaccine compared to other constructs of the HBM, supporting hypothesis 1. This underscores that the greater the perceived susceptibility, the higher the individual’s likelihood of getting the vaccine, which would mitigate the risks of COVID-19 exposure. The possible reasons for this may be related to the ‘hypochondriac concerns” (worry about being infected and infecting family) and the panic that the disease would be difficult to control. In a similar sense, Dini et al. [36] argued that when self-protection beliefs are associated with protecting others, the altruistic behaviors of the population are reduced, and vaccine uptake is higher. However, Wong et al. [19] found that perceived susceptibility was not associated with vaccine acceptance.

On the other hand, the findings of our study also revealed that the perceived severity of COVID-19 has the most significant influence on the intention to receive a vaccine. This implies that people who perceive COVID-19 as serious will be more likely to accept the vaccine. From a psychological perspective, individuals take actions toward health if they believe that harm can be serious [37]. The severity of the health risk is a key feature in convincing people to get vaccinated. In fact, the date our survey questionnaire was finished, COVID-19 caused more than two million confirmed cases and about 157955 deaths worldwide [38].This health threat is assumed as an indication to alter the public’s future behavior toward a vaccine against COVID-19. Musa Ibrahim discussed that severity includes beliefs about the disease itself and broader impacts of the disease on an individual’s social role [39]. This, in turn, may lead to higher chances of behavior to avoid these impacts. Transmission dynamic models of the COVID-19 outbreak show that containment played a major role in the spread limitation of infection in Morocco [40]. Taking this into account, the promotion of extension services and public awareness campaigns to sensitize the public as to the persistent risk of COVID-19 even after de-containment can be regarded as a key policy option [41]. In this respect, educational courses highlighting personal risks to the disease and using fear appeal messages via mass media such as television and social media are defined as making the public aware of the severity of coronavirus and promoting the COVID-19 vaccine.

Our findings partially supported Hypothesis 2, as the only perceived benefit was positively associated with the intention to receive the COVID-19 vaccine. However, we suggest a negative association between perceived barriers and the acceptance of the COVID-19 vaccine. In our study, the general public took a generally favorable opinion toward COVID-19 vaccines. They believed the vaccines effectively prevented the disease for both themselves and their families. Nevertheless, long-term side-effects, inadequate data concerning the COVID-19 vaccine, fear of the vaccine causing the COVID-19 disease, and confusion about efficacy were serious barriers to people’s vaccination intention. This finding is in concordance with previous studies conducted in other regions, showing that beliefs about COVID-19 vaccines’ benefits and barriers influenced intention to receive COVID-19 vaccine; the former was positive, while the latter was negative [15,42]. Overall, lack of knowledge of the COVID-19 vaccine and reservations about side-effects are still the key reasons for hesitancy to get the vaccine [29]. In Morocco, the safety, efficacy, and strengths of the COVID-19 vaccine adopted have been confirmed through positive findings and clinical trials conducted in Morocco and other countries [4]. Thus, building trust between the Moroccan population and health authorities, disseminating correct knowledge, and ensuring transparency in the process of vaccine approval through an effective outreach strategy could be an effective option to improve vaccine acceptance. Furthermore, public health authorities need to inform the public about expected post-vaccination symptoms as well as the rarity of major side effects.

Different from existing research [18], this study’s findings showed a significant positive association between CtA and self-efficacy and intention to get vaccinated for COVID-19, supporting Hypothesis 3. This is partially consistent with previous research on H1N1, which also shows that self-efficacy is positively related to vaccine uptake [43]—knowing that getting vaccinated to prevent contracting COVID-19 may motivate the public to adopt the behavior. Thus, the recommendation from the expert and doctor stood out as the most important cue, far stronger than others, such as from friends and family members. Therefore, since our result reveals that people have trust in health authorities and they agree to follow the advice of their doctor, the healthcare professionals should be equipped with the knowledge of vaccine development, efficacy, and provide information about the difference between selected vaccines so that they can confidently answer the concerns of the public and make them understand the rationale for getting vaccinated against COVID-19.

Our study has several limitations that can be addressed in future work. First, considering the limited resources available and the time sensitivity of the COVID-19 outbreak, we followed the snowball sampling method. The snowballing sampling technique did not use a random sample range, and the study population did not represent the general population’s actual trend. Secondly, our study was limited in scope. Our sample was obviously over-representative of men and well-educated people, limiting the generalization of our findings to the whole population. Thirdly, this study was carried out before COVID-19 vaccines were available. Thus, actual vaccine uptake was not evaluated. A further follow-up to test the current framework at a later pandemic stage could give a complete picture of vaccine hesitancy. Fourth, although incorporating elements affecting the intention to get vaccinated against COVID-19 in the model significantly improved the model’s explanatory capacity, a considerable part of the variance remained unexplained. Future research could include other variables such as attitudes toward vaccines, fears, and injunctive norms that have been relatively less examined.

As of 6 March 2022, fortunately, against the findings of this study, 14 months after the survey was conducted in January 2021, the percentage of Moroccans who had received at least one and two vaccination shots were 66.59% and 62.47%, respectively [44], exceeding the levels predicted by the outcome of this study analysis. Thus, the reason why the Moroccan community was able to overcome its initial low vaccination intentions and achieve a high vaccination achievement rate remains an issue to be unraveled by future research and constitute the last limitation of this study.

Despite these above limitations, the findings from this study have important public health implications. Overall, the findings indicate that our ability to increase uptake of available COVID-19 vaccines among females, young adults and participants with lower education levels may rely on appropriately communicating information specifically about the safety of the vaccine, how it works to protect against infection, and why it is important to protect oneself and others against COVID-19. Given the public’s confidence in an expert or doctor, it will be necessary for public health strategies to engage healthcare professionals as possible communication channels for trusted information. These strategies could be successful in increasing inoculation uptake. It is also essential to implement strategies to limit the spread of conspiracy theories.

## 5. Conclusions

This study is the first in Morocco to shed the light on the socio-psychological factors that determine Moroccan’s intention to receive the COVID-19 vaccine. Overall, various demographic and psychological predictors were correlated to the intention to get vaccinated against COVID-19. The results showed that perceived susceptibility and benefits were the strongest predictors of acceptance of the COVID-19 vaccine. Further, given the negative correlation between perceived barriers and vaccination intention, it is necessary to maintain a high level of transparency regarding the vaccines’ effectiveness and safety. Our study findings raise major concerns about equitable vaccination, with lower acceptance among individuals with chronic disease who most require the vaccinations. There is an urgent need to further explore and address the fears and concerns of these groups to ensure equitable access to and utilization of COVID-19 vaccines. These identified constructs provide evidence-based formulation and implementation of vaccination strategies to bolster the overall COVID-19 immunization program.

## Figures and Tables

**Table 1:** Frequency of intention to receive COVID-19 vaccine by sociodemographic characteristics.

Characteristics	Total Number (%)	I Intend to Get Vaccinated	Not Intending to Vaccinate	*p*-Value
**Total, No (%)**	3800 (100)	1521 (40)	2279 (60)	
**Gender**				
Men	2175 (57.2)	829 (38.1)	1346 (61.8)	0.003
Women	1625 (42.8)	692 (42.6)	933 (57.4)	
**Age group**				
18–29	806 (21.2)	36 (42.9)	460 (57.1)	0.046
30–44	1690 (44.5)	661 (39.1)	1029 (60.9)	
45–59	1114 (29.3)	430 (38.6)	684 (61.4)	
>60	190 (5)	84 (44.2)	106 (55.8)	
**Marital status**				
Others	1693 (44.6)	617 (36.4)	1076 63.6)	0.000
Married	2107 (55.4)	904 (42.9)	1203 (57.1)	
**Educational level**				
>Primary school	617 (16.2)	228 (37)	389 (63)	0.033
Secondary school	1003 (26.4)	377 (37.6)	626 (62.4)	
Bachelor’s degree	963 (25.3)	409 (42.5)	554 (57.5)	
Master’s and PhD	1217 (32)	507 (41.7)	710 (58.3)	
**Chronic disease**				
None	2439 (64.2)	1000 (41)	1439 (59)	0.041
Present	1361 (35.8)	521 (38.7)	840 (61.7)	
**Influenza vaccine**				
Never	3271 (86.1)	1334 (40.8)	1937 (59.2)	0.01
Once a year or more	529 (13.9)	187 (35.3)	342 (64.7)	
**Infection with COVID-19**				
Not infected	2171 (57.1)	837 (38.6)	1334 (61.4)	0.019
Confirmed cases	1629 (42.9)	684 (42.0)	945 (58.0)	
**Monthly family income (MAD)**				
<2000,	1198 (31.5)	510 (42.6)	688 (57.4)	0.043
2000–4000,	197 (5.2)	74 (37.6)	123 (62.4)	
4000–8000,	449 (11.8)	178 (39.6)	271 (60.4)	
8000–12,000,	921 (24.2)	364 (39.5)	557 (60.5)	
>12,000	1035 (27.2)	395 (38.5)	640 (61.5)	

*p*-value for the Pearson chi-squared proportion test at significance level of (α) 5%.

**Table 2:** Factors associated with acceptance of COVID-19 vaccine by binary logistic regression.

Total, No (%)	n	Vaccine Acceptance %	Odds Ratios	95% Confidence Interval	*p*-Value
**Gender**					
Men	2175	38.1	Ref		
Women	1625	42.6	1.24	1.09–1.42	0.001
**Age group**					
18–29	806	42.9	ref		
30–44	1690	39.1	1.19	1.05–1.67	0.030
45–59	1114	38.6	1.27	1.13–1.68	0.023
>60	190	44.2	1.32	1.18–1.74	0.047
**Marital status**					
Others	1693	36.4	ref		
Married	2107	42.9	1.31	1.16–1.50	0.000
**Educational level**					
>Primary school	617	37	ref		
Secondary school	1003	37.6	0.98	0.82–1.16	0.831
Bachelor’s degree	963	42.5	1.18	0.99–1.40	0.055
Master’s and PhD	1217	41.7	1.21	0.99–1.48	0.059
**Chronic disease**					
None	2439	41	ref		
Present	1361	38.3	1.24	1.09–1.62	0.004
**Influenza vaccine**					
Never	3271	40.8	ref		
Once a year or more	529	35.3	1.07	0.93–1.23	0.322
**Infection with COVID-19**					
No infected	2171	38.6	ref		
Confirmed cases	1629	42	1.20	1.05–1.38	0.007
**Monthly income family (MAD)**					
<2000,	1198	42.6	ref		
2000–4000,	197	37.6	0.83	0.69–1.02	0.078
4000–8000,	449	39.6	1.05	0.76–1.444	0.774
8000–12,000,	921	39.5	0.96	0.76–1.21	0.727
>12,000	1035	38.3	0.95	0.80–1.15	0.632

**Table 3:** Descriptive statistics of key variables concerning COVID-19 vaccine acceptance (*n* = 3800).

Construct	Variable Description (Symbols)	Mean (SD)
**Perceived susceptibility (Sus)**		3.90 (1.22)
	I am at risk of getting COVID-19 (Suc1)	3.88 (1.3)
	It is likely that my children will be infected by the Coronavirus (Suc2)	3.92 (1.19)
	It is possible that the elderly will get COVID-19 in the coming 9 months (Suc3)	3.92 (1.18)
Cronbach’s α		0.778
**Severity of COVID-19 (Sev)**		3.28 (1.23)
	I think that COVID-19 is a serious threat to human health (Sev1)	4.12 (1.05)
	I believe that if I catch COVID-19 it will have a serious consequence for my life (Sev2)	2.42 (1.21)
	I believe that COVID-19 can lead to death of my loves one if they get infected (Sev3)	2.97 (1.36)
	I’m afraid to catch COVID-19 (Sev4)	3.62 (1.32)
Cronbach’s α		0.703
**Perceived barriers (Bar)**		4.53 (0.63)
	I have concerns about COVID-19 vaccine long-term side effects (Bar1)	4.78 (0.57)
	Not enough research done about COVID-19 vaccine (Bar2)	4.45 (0.64)
	The COVID-19 vaccine causes a person to get COVID-19 (Bar3)	4.45 (0.66)
	I am not sure if COVID-19 vaccine is effective in preventing the disease (Bar4)	4.45 (0.64)
Cronbach’s α		0.701
**Perceived benefits (Ben)**		4.5 (0.82)
	COVID-19 vaccine will be effective in preventing Coronavirus (Ben1)	4.67 (0.82)
	If I get the vaccines, I will be less likely to get COVID-19 (Ben2)	4.57 (0.83)
	I think COVID-19 vaccine can prevent people from spreading the virus to others (Ben3)	4.27 (0.82)
Cronbach’s α		0.665
**Self-efficacy (SE)**		4.5 (0.84)
	I will be able to get the vaccines to prevent contracting COVID-19 (SE1)	4.76 (0.66)
	It will be easy for me to get the vaccines to protect myself from COVID-19 (SE2)	4.28 (0.87)
	Getting vaccinated to prevent COVID-19 is convenient (SE3)	4.47 (1.00)
Cronbach’s α		0.763
**Cues to action (CtA**)		4.31 (0.89)
	I will be more optimistic about COVID-19 vaccine if I know more about it (CtA1)	4.05 (0.96)
	The type of vaccine that is available would affect my decision (CtA2)	4.39 (0.94)
	I would be more confident if experts or people I trust would recommend the vaccine (CtA3)	4.49 (0.76)
Cronbach’s α		0.668
**Intention to receive a COVID-19 vaccine (Int)**		3.56 (1.28)
	I intend to get vaccinated as soon as possible (Int1)	3.49 (1.39)
	I probably get it but not as soon as possible (Int2)	3.84 (1.19)
	I get vaccinated if an expert or doctor I trust recommend me COVID-19 vaccines (Int3)	2.74 (1.41)
	I am currently undecided (Int4)	4.18 (1.16)
Cronbach’s α		0.718

**Table 4:** Results of first-order confirmatory factor analysis.

Constructs	Measurement Items	Std. Loading	t-Value	Reliability and Variability
**Perceived susceptibility (Sus)**	Sus1	0.281	29.579	AVE = 0.656; CR = 0.828; ASV = 0.017; MSV = 0.033
	Sus2	0.977	93.517	
	Sus3	0.963	fixed	
**Severity of COVID-19 (Sev)**	Sev1	0.856	16.052	AVE = 0.502; CR = 0.789; ASV= 0.024; MSV = 0.062
	Sev2	0.36	17.342	
	Sev3	0.721	fixed	
	Sev4	0.7	23.626	
**Perceived barriers (Bar)**	Bar1	0.681	29.601	AVE = 0.509; CR = 0.794; ASV = 0.025; MSV = 0.098
	Bar2	0.391	21.988	
	Bar3	0.859	fixed	
	Bar4	0.826	51.166	
**Perceived benefits (Ben)**	Ben1	0.839	fixed	AVE = 0.524; CR = 0.740; ASV = 0.026; MSV = 0.171
	Ben2	0.887	32.896	
	Ben3	0.289	16.638	
**Self-efficacy (SE)**	SE1	0.825	20.785	AVE = 0.548; CR = 0.779; ASV = 0.051; MSV = 0.098
	SE2	0.563	16.638	
	SE3	0.804	fixed	
**Cues to action (CtA)**	CtA1	0.613	27.509	AVE = 0.528; CR = 0.767; ASV = 0.030; MSV = 0.171
	CtA2	0.688	31.806	
	CtA3	0.858	fixed	
**Intention (Int)**	Int1	0.8	fixed	AVE = 0.504; CR = 0.866; ASV = 0.051; MSV = 0.171
	Int2	0.666	26.623	
	Int3	0.759	20.535	
	Int4	0.828	23.626	

## Data Availability

Not applicable.
